# Relapsing *Campylobacter jejuni* Systemic Infections in a Child with X-Linked Agammaglobulinemia

**DOI:** 10.1155/2013/735108

**Published:** 2013-06-03

**Authors:** Paola Ariganello, Giulia Angelino, Alessia Scarselli, Irene Salfa, Martina Della Corte, Arianna De Matteis, Patrizia D'Argenio, Susanna Livadiotti, Emma C. Manno, Cristina Russo, Andrea Finocchi, Caterina Cancrini

**Affiliations:** ^1^University-Hospital Department of Pediatrics, Unit of Immunology and Infectious Disease, Children's Hospital Bambino Gesù and University of Rome “Tor Vergata”, Rome, Italy; ^2^Department of Laboratories, Unit of Microbiology, Children's Hospital Bambino Gesù, Piazza Sant'Onofrio, 4-00165 Rome, Italy

## Abstract

X-linked agammaglobulinemia (XLA) is a primary immunodeficiency of the humoral compartment, due to a mutation in the *Bruton tyrosine kinase (BTK)* gene, characterized by a severe defect of circulating B cells and serum immunoglobulins. Recurrent infections are the main clinical manifestations; although they are especially due to encapsulated bacteria, a specific association with *Campylobacter* species has been reported. Here, we report the case of a boy with XLA who presented with relapsing *Campylobacter jejuni* systemic infections. His clinical history supports the hypothesis of the persistence of *C. jejuni* in his intestinal tract. Indeed, as previously reported, XLA patients may become chronic intestinal carriers of *Campylobacter*, even in absence of symptoms, with an increased risk of relapsing bacteraemia. The humoral defect is considered to be crucial for this phenomenon, as well as the difficulties to eradicate the pathogen with an appropriate antibiotic therapy; drug resistance is raising in *Campylobacter* species, and the appropriate duration of treatment has not been established. *C. jejuni* should always be suspected in XLA patients with signs and symptoms of systemic infection, and treatment should be based on antibiogram to assure the eradication of the pathogen.

## 1. Introduction

X-linked agammaglobulinemia (XLA) is a primary immunodeficiency characterized by an abnormal development of B lymphocytes and severe hypogammaglobulinemia, due to a mutation in the *Bruton Tyrosine Kinase* (*BTK*) gene. *Campylobacter* is responsible for persistent infections in XLA patients [1]. We report the case of a boy with XLA who presented with relapsing *Campylobacter jejuni* systemic infections.

## 2. Case Report

A one-year-old boy admitted for severe *Staphylococcus aureus* impetigo and sepsis was diagnosed with XLA (missense mutation of *BTK* gene 1706 G>C, R525P; absent expression of *BTK* protein from western blot analysis). Treatment with intravenous immunoglobulins (IVIGs) every 28 days was started. Followup had been uneventful until the age of 8 when he was admitted for painful swelling and hyperemia of the left knee, suggestive for a cellulitis. Anamnesis was negative for recent infections or trauma. A knee ultrasound revealed a small intra-articular fluid collection. Blood exams showed neutrophilic leukocytosis with normal. C-reactive protein and IgG level was 807 mg/dL. Microbiological investigations could not be performed. Empiric treatment with piperacillin/tazobactam was promptly started, with a rapid resolution of symptoms. During admission, the child presented several self-limiting episodes of diarrhea. He was discharged in good condition.

At the age of 11, the boy was admitted for an episode of sepsis, characterized by high fever and acute enteritis with dehydration. White blood cell count was increased (with neutrophils 85%), as well as C-reactive protein. IgG level was 684 mg/dL. Empiric treatment with ceftriaxone was rapidly effective, and the child was discharged after few days, with intramuscular antibiotic therapy. The results of microbial cultures performed on blood and stool revealed the presence of *Campylobacter jejuni *spp*. jejuni*, in both specimens collection, having the same antimicrobial susceptibility pattern, resistance to quinolones but a sensitivity to macrolides (ceftriaxone was not tested).

**Figure 1:**
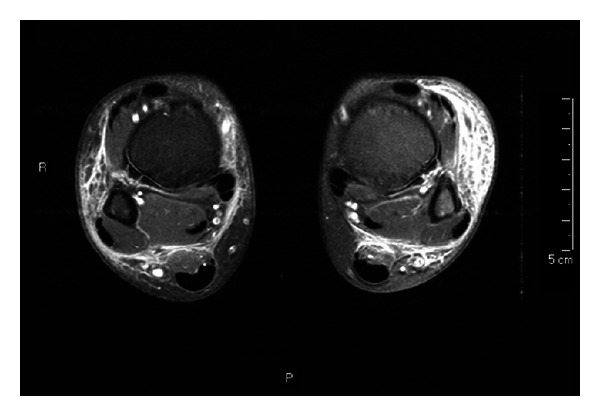
Magnetic resonance imaging of ankles and legs (coronal T2 and axial T1) showing bilateral cellulitis. Images show an extensive signal alteration of the subcutaneous soft tissues in both legs with dishomogeneous enhancement after contrast, consistent with an inflammatory involvement (cellulitis). A bilateral small intra-articular collection can be seen, without significant enhancement after contrast. Neither alterations in muscles and tendons, nor signs of osteomyelitis are detectable.

At the age of 12, the boy had a gastrocnemius muscle tear after a trauma on his right ankle, confirmed by ultrasound and treated with compression bandage. In this period, he occasionally presented with swelling of the other ankle, ascribed to difficulty of walking. After bandage removal, both ankles and feet showed a worsening swelling with pain and fever, partially responsive to nonsteroidal anti-inflammatory drugs and ciprofloxacin. Because of the persistence of bilateral ankle edema and supramalleolar ecchymosis with hot and erythematous legs, the patient was admitted to our hospital. A neutrophilic leukocytosis was detected, inflammation indices were increased, and IgG level was 638 mg/dL. Ultrasound and magnetic resonance imaging confirmed a picture of bilateral cellulitis of ankles and legs (Figure 1). Involvement of joints, tendons, muscles, and bones was excluded, as well as thrombophlebitis. Suspecting an infection by encapsulated bacteria, combined empiric antibiotic therapy was started with intravenous vancomycin and oral ciprofloxacin, in addition to the infusion of IVIG 400 mg/kg. Fever persisted although partial response was observed with progressive reduction of the swelling. Although stool culture was negative, *C. jejuni* spp.* jejuni grew* in three consecutive blood cultures. Therefore, therapy was changed to meropenem and clarithromycin on the basis of the antibiogram result; indeed the microorganism was resistant to quinolones and ceftriaxone but sensible to macrolides. A progressive improvement of skin lesions and clinical conditions was observed, with normalization of inflammation markers, and supported by two subsequent negative blood cultures. The child was discharged after 10 days with oral clarithromycin home therapy for 3 weeks. Followup has been uneventful for the next 12 months with negative stool cultures.

## 3. Discussion

*Campylobacter jejuni *and* coli* are the most common pathogens in humans' fecal cultures. Unlike the closely related organism *C. Fetus*, *C. jejuni *is not frequently associated with bacteraemia and is responsible for self-limiting gastrointestinal infections in immunocompetent subjects. Nevertheless, a decreased immune response, as it may also occur in elderly people or immunocompromised patients (i.e., immunodeficiency, HIV infection, diabetes, cirrhosis, cancer, and chemotherapy), increases the risk of developing a severe infection from this pathogen. A specific association is known between XLA and *Campylobacter *systemic infections [1], such as bacteraemia, cellulitis, osteomyelitis, arthritis, pericarditis and endocarditis [2]. Likewise, patients with common variable immunodeficiency are more susceptible to *C. jejuni* infections, especially those with undetectable IgA [3]. Only few pediatric cases of *Campylobacter* bacteraemia have been described in the literature [2, 4]; such a paucity of cases may be due to diagnostic bias (insufficient blood samples, lack of subcultures) or even to the absence of blood cultures in the diagnostic workup.

Our XLA patient presented with two infections, in which the presence of *C. jejuni* was microbiologically confirmed. In one circumstance, gastroenteritis and sepsis were the predominant clinical findings, while the second episode was characterized by cellulitis of both ankles and legs without intestinal symptoms. Because of the suggestive clinical picture of the first episode of cellulitis of the left knee (involvement of soft tissues in addition to diarrhea), we can speculate that it could be due to the same microorganism. Our patient's clinical history supports the hypothesis of the persistence of *C. jejuni* in the intestinal tract, with relapsing systemic infections [4]. Interestingly, this phenomenon has been demonstrated by cultures of biopsy specimens from intestinal mucosa of XLA patients despite negative stool cultures [4]. Immunoglobulins defect is considered to be crucial; although protective levels of IgG are provided by IVIG infusions, IgA and IgM are persistently decreased; IgA plays an important role in the defense against *C. jejuni* in the gastrointestinal mucosa, while IgM has been shown to contribute to the serum bactericidal activity against this pathogen [3]. An additional risk factor for persistence and relapses of *Campylobacter* infections consists of the difficulties to eradicate the pathogen with an appropriate antibiotic therapy [2]. The length of the specific treatment could represent a crucial aspect to prevent relapses. Furthermore, resistance to antimicrobial drugs is increasing; quinolones should not be chosen for empirical treatment, while sensitivity to macrolides, which are still the treatment of choice, should be verified by antibiogram [5]. Obviously, the extensive use of antibiotic prophylaxis in XLA patients (mainly for respiratory infections) may contribute to an increased risk of resistance of *Campylobacter* species, as well as the use of empiric anti-infective treatments. Therefore, prophylactic and empiric antibiotic therapies should always be carefully assessed in these patients.

In conclusion, blood and stool cultures for *C. jejuni* should always be performed in XLA patients with signs and symptoms of systemic infection. To assure the eradication of the pathogen and prevent relapses, treatment should be administrated on the basis of the antibiotic susceptibility test, because of the high resistance of *C. jejuni *to several common antibiotics [5]. A prolonged treatment may be carefully considered, and stool cultures should be regularly performed during followup even in the absence of symptoms.

We also suggest, as surveillance procedure, to perform stool cultures for *C. jejuni* as a screening in all XLA patients in order to identify asymptomatic carriers, although treatment is questionable in absence of symptoms. Furthermore, it should be kept in mind that negative cultures do not exclude gastrointestinal colonization.
